# Supplementary material for: Low-Cost Automated Vectors and Modular Environmental Sensors for Plant Phenotyping
Source: Sensors (Basel). 2020 Jun 11;20(11):3319. doi: 10.3390/s20113319 (PMC7309146; doi:10.3390/s20113319)

Figure S1. Thermal Imager wiring schematic

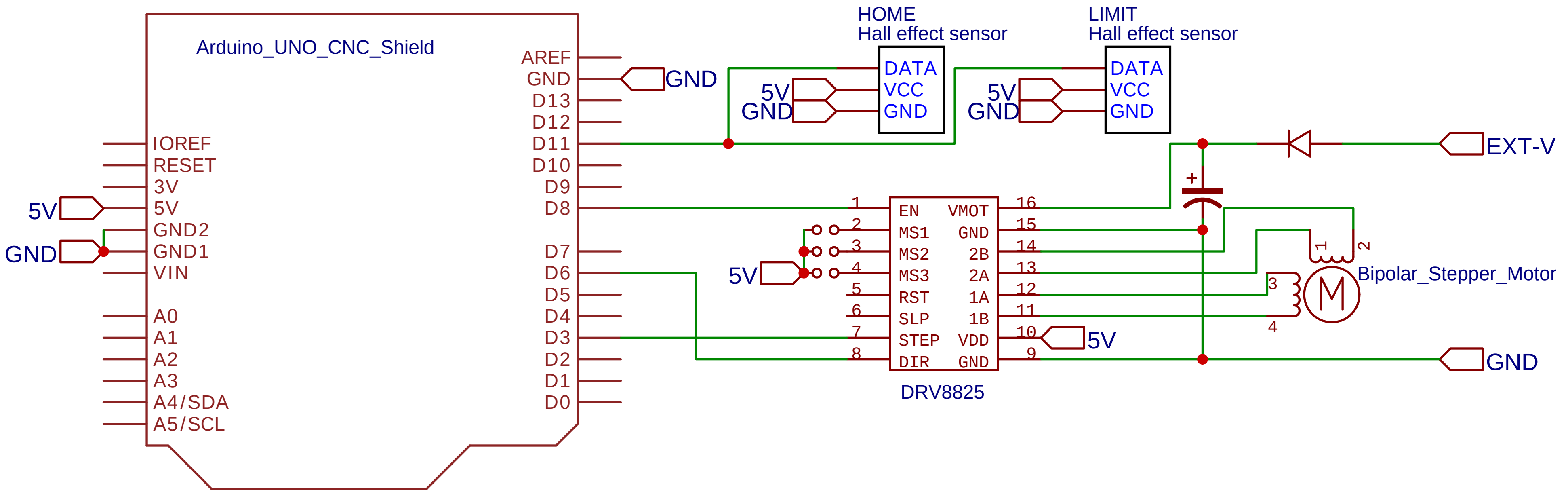

Figure S2. Plate Imager wiring schematic

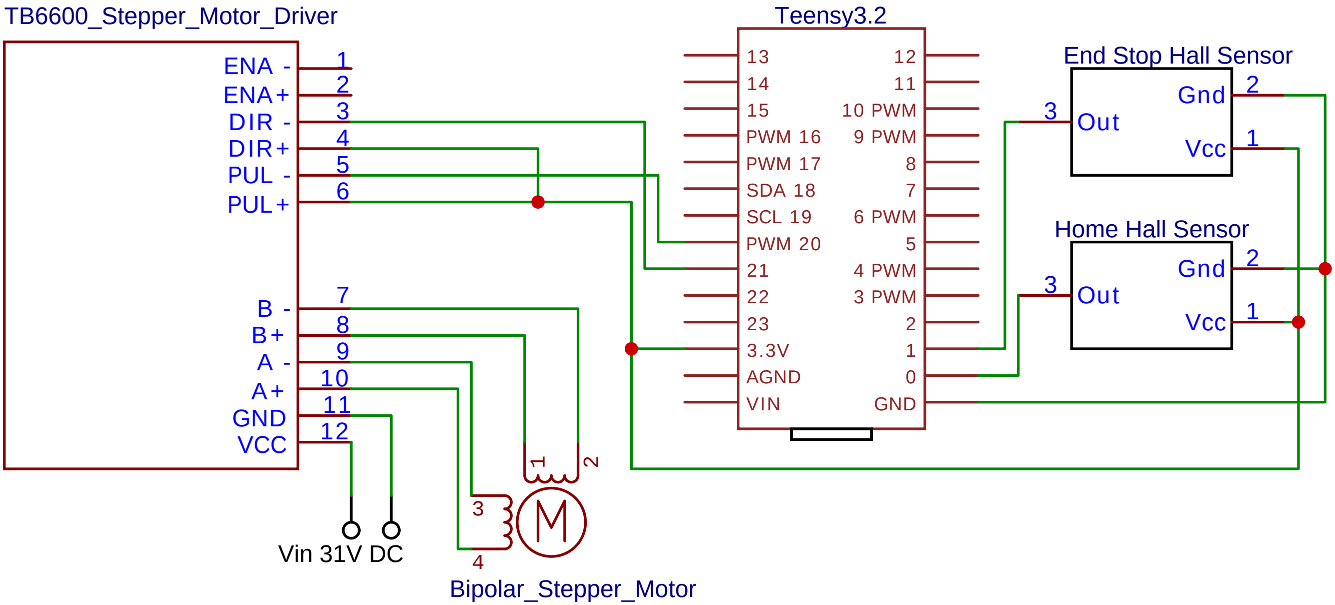

Figure S3. Sensor platform PCB schematic

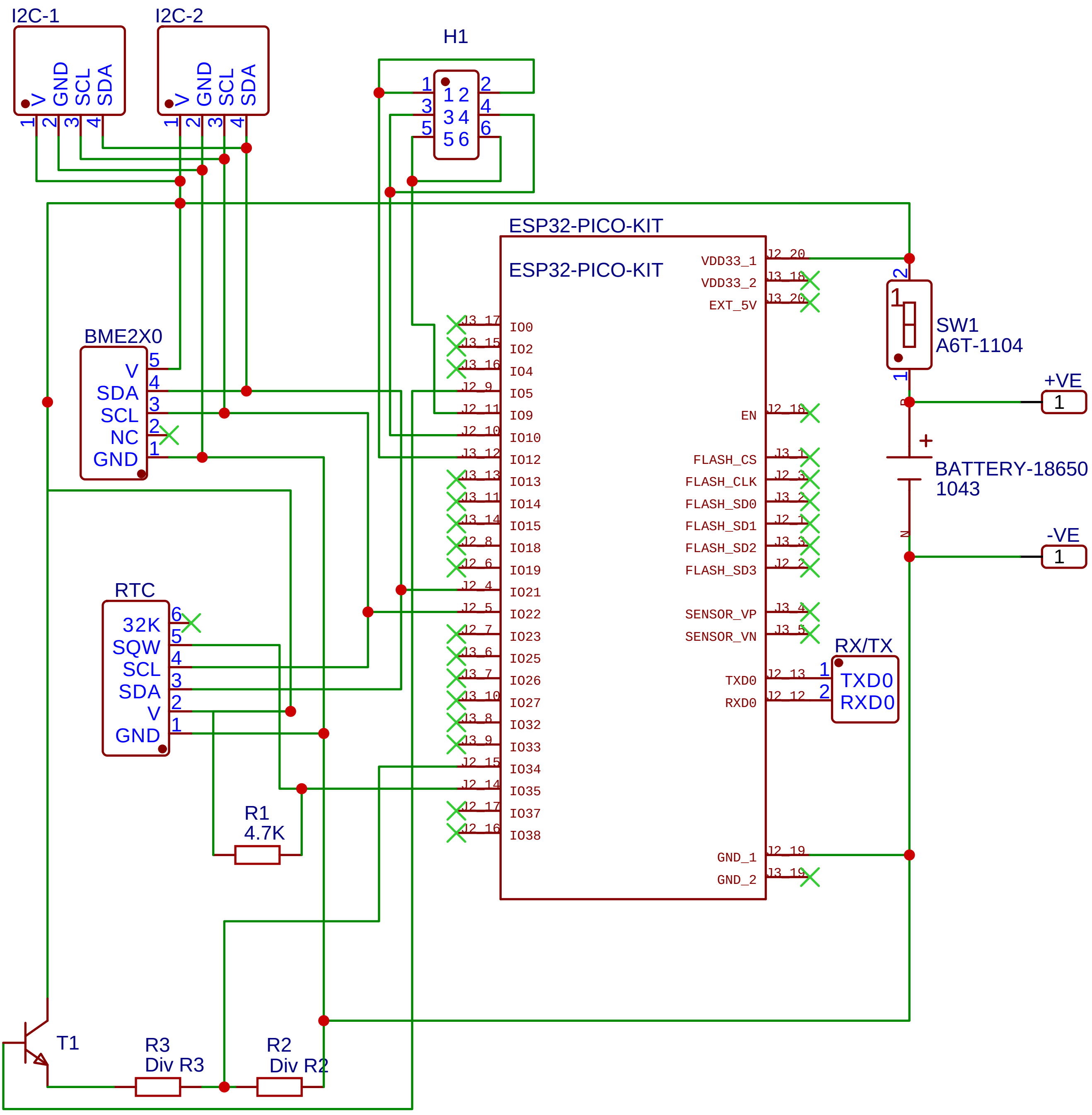

Supplement: Supplementary file 1 [file sensors-20-03319-s001.pdf]
